# Can you eat it? A link between categorization difficulty and food likability

**DOI:** 10.2478/v10053-008-0120-2

**Published:** 2012-08-21

**Authors:** Yuki Yamada, Takahiro Kawabe, Keiko Ihaya

**Affiliations:** 1 The Research Institute for Time Studies, Yamaguchi University, Japan; 2 Faculty of Human-Environment Studies, Kyushu University, Japan; 3 Japan Society for the Promotion of Science; 4 NTT Communication Science Laboratories, Japan

**Keywords:** categorization, food neophobia, appetite, emotion

## Abstract

In the present study we examined whether categorization difficulty regarding a food is related to its likability. For this purpose, we produced stimulus images by morphing photographs of a tomato and a strawberry. Subjects categorized these images as either a tomato or a strawberry and in separate sessions evaluated the food’s eatability or the subject’s willingness to eat (Experiments 1 and 2) and the likeliness of existence of each food (Experiment 2). The lowest score for ca- tegorization confidence coincided with the lowest scores for eatability, willingness to eat, and likeliness of existence. In Experiment 3, we found that food neophobia, a trait of ingestion avoidance of novel foods, modulated food likability but not categorization confidence. These findings suggest that a high categorization difficulty generally co-occurs with a decrease in food likability and that food neophobia modulates likability. This avoidance of difficult-to-categorize foods seems ecologically valid because before eating we have little information regarding whether a food is potentially harmful.

## Introduction

In daily life, we categorize various objects, people, and events into appropriate categories (e.g., “It is a fruit”; “He is Japanese”; or “This story is a lie”). Appropriate categorization is essential for adaptive life; if we cannot correctly categorize an object as safe or dangerous, we can neither avoid the danger nor reach safety. It is known that we feel a negative impression of an object if we find it difficult to categorize (Yamada, Kawabe, & Ihaya, in press). Yamada et al. showed that cate-gorization difficulty is related to the uncanny valley phenomenon, in which human-like robots sometimes elicit unpleasant impressions of human observers who watch the robots, such as eeriness and disgust (Mori, 1970). Yamada et al. morphed two images of real, cartoon, or stuffed human facial images. Subjects were then asked to categorize the stimulus images and to evaluate the likability of each face. The results showed that likability decreased when categorization was difficult. They obtained similar results when using stimulus images created by morphing images of different dogs instead of human facial images, suggesting that this effect was not stimulus-specific. These results were interpreted as indicating that categorization difficulty of an object is closely linked to its likability.

The effect of categorization difficulty on likability evaluation of objects has been tested in terms of both human and animal face stimuli. A different stream of research indicated category-specific semantic deficits and suggested the existence of distinct mechanisms to categorize living and non-living things (Forde & Humphreys, 2002). However, it remains unclear whether categorization difficulty for non-living things is related to their likability.

The present study was performed to examine the effects of categorization difficulty on likability of food. If categorization difficulty is closely linked to object likability regardless of its animacy, then it is expected that a high categorization difficulty for a food will co-occur with a decrease in its likability.

In addition, it is necessary to consider effects of individual differences regarding food likability. *Food neophobia* is an ingestion-avoidance response toward novel foods, and is considered to be a characteristic of omnivores, such as humans (Pliner & Salvy, 2006). Food neophobia is deemed to serve a protective function to prevent the ingestion of potentially harmful foods by evoking negative emotional reactions to unfamiliar foods. In experimental research, Pliner, Pelchat, and Grabski (1993) found that observers rated novel foods to be more dangerous than familiar foods, and willingness to eat was related to disliking and dangerousness. There is much evidence that top-down information reduces food neophobia, indicating the involvement of higher cognitive processing (Harper & Sanders, 1975; Martins, Pelchat, & Pliner, 1997; McFarlane & Pliner, 1997; Tuorila, Meiselman, Bell, Cardello, & Johnson, 1994). Moreover, food neophobia seems to be irrelevant to low-level image properties that affect the perception of food freshness (Péneau, Brockhoff, Escher, & Nuessli, 2007; Wada et al., 2010) because food neophobia occurs even with fresh foods. In this way, several aspects of food neophobia have been clarified. However, it is still unclear how negative emotional reactions associated with food neophobia are related to a decrease in likability due to high categorization difficulty.

The first aim of the present study was to ascertain whether categorization difficulty is related to food likability. We used eatability (suitability for use as a food) and willingness to eat as indices of food likability, and predicted that high categorization difficulty will co-occur with decreases in both indices.^1^ The second aim was to investigate how food neophobia is related to the decrease in food likability associated with high categorization difficulty. Pliner and Hobden (1992) developed a scale to measure food neophobia, and found that there were individual differences in the trait. We predict that individual differences in food neophobia will affect categorization and food likability if food neophobia is related to categorization difficulty. Alternatively, food neophobia may only influence food likability if it is irrelevant to the categorization difficulty of a food. Here, we measured the degree of food neophobia in each subject using a questionnaire, and examined how individual differences in food neophobia influence the categorization and likability of foods.

## Experiment 1

### Methods

#### Subjects, apparatus, and stimuli

A total of 21 subjects (nine women, 12 men; M_age_=23.57 years, *SD* = 4.30) participated in this experiment, and each received a payment of Ą500 (approx. US$5.00). Eleven were assigned to the eatability condition and 10 to the willingness to eat condition. All subjects were naive as to the purpose of the present study, and all reported that they had normal or corrected-to-normal visual acuity.

The stimuli were presented on a 19-inch CRT monitor (RDF193H; Mitsubishi, Tokyo, Japan) with a resolution of 1,024×768 pixels, and a refresh rate of 100 Hz. The presentation of stimuli and the collection of data were controlled by a computer (Mac Pro; Apple, Cupertino, CA).

**Figure 1.**
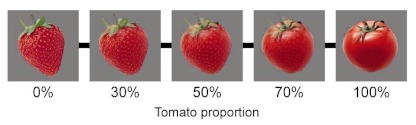
Examples of stimuli used in this study.

Stimuli consisted of a fixation point, command cursors for rating, and images of morphed tomato and strawberry photographs (Figure 1). Stimulus size was provided in visual angles at a viewing distance of 40 cm. The fixation point was composed of two concentric rings, one small and one large, with radii of 0.24° and 0.47°, respectively. The luminance of each ring was 91.0 cd/m^2^. The command cursors were white boxes surrounding each rating value (0.95×1.89°; 91.0 cd/m^2^) and a selected box was filled in white. We employed color pictures (12.1×12.1°) of a tomato and a strawberry. We generated 11 equally stepped morphed images with morphing proportions ranging from 0 to 100%. Each stimulus was displayed on a gray background (43.5 cd/m^2^).

#### Procedure

The experiment was conducted in a darkened room. The subject’s visual field was fixed using a chin headrest, at a viewing distance of 40 cm. The experiment consisted of two task blocks: a categorization task and an evaluation task. The order of the blocks was counterbalanced across the subjects.

Each subject initiated each trial by pressing the spacebar on a computer keyboard. The fixation point was presented throughout the experiment whenever the image was not on-screen. In each trial, after a delay of 500 ms, a morphed image was presented and remained on the screen until the subject’s response. In the categorization task, the subjects were asked to categorize the food in the morphed image as tomato or strawberry using a 7-point scale ranging from -3 (*definitely a tomato*) to 3 (*definitely a strawberry*) by pressing selection keys and a decision key. We used the absolute value of this categorization score as “categorization confidence.”

In the evaluation task, the subjects in the eatability condition were asked to evaluate the eatability of each food using a 7-point scale ranging from -3 (*definitely uneatable*) to 3 (*definitely eatable*). In contrast, the subjects in the willingness to eat condition were asked to evaluate their willingness to eat each food using a 7-point scale ranging from -3 (*I never want to eat it*) to 3 (*I strongly want to eat it*). Rapid responses were not encouraged. Each subject performed 22 trials with 11 images and two tasks. The trial order was randomized for each subject.

### Results and discussion

**Figure 2.**
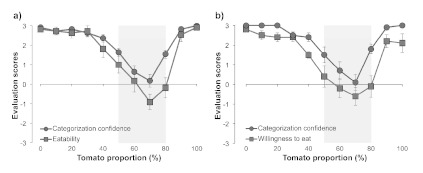
Results of Experiment 1 in the (A) eatability and (B) willingness to eat conditions. The gray areas indicate the tomato proportions in which both eatability or willingness to eat and categorization confidence were significantly lower than those in both the tomato proportions of 0% and 100%. Error bars denote the standard errors of the mean.

Figure 2 shows the results of Experiment 1. For the results of the eatability condition, one-way analysis of variance (ANOVA), with the Tomato Proportion in the morphed images as a factor, performed on categorization confidence showed a significant main effect, *F*(10, 100)=30.57, *p*<.0001. Multiple comparisons using Ryan’s (1960) method revealed that categorization confidence for 50-80% images was significantly lower than for both 0% and 100% images (*p*s<.0001). One-way ANOVA on the eatability score revealed a significant main effect of the tomato proportion, *F*(10, 100)=22.76, *p*<.0001. Multiple comparisons revealed that the eatability score for 50-80% images was significantly smaller than for both 0% and 100% images (*p*s<.0001). One-way ANOVA on categorization confidence revealed a significant main effect of the tomato proportion on the willingness to eat, *F*(10, 90)=19.86, *p*<.0001. Multiple comparisons indicated that the categorization confidence for 50-80% images was significantly lower than for both 0% and 100% images (ps < .0001). ANOVA on willingness to eat score revealed a significant main effect of the tomato proportion, *F*(10, 90)=16.08, *p*<.0001. Multiple comparisons revealed that the willingness to eat score for 50-80% images was significantly smaller than for both 0% and 100% images (*p*s < .0001).

We calculated the points of minimum categorization confidence and the minimum eatability (or willingness to eat) score and their 95% confidence intervals by fitting a Gaussian function to mean categorization confidence and mean eatability (or mean willingness to eat) score as a function of the tomato proportion. For the eatability condition, comparisons using 95% confidence intervals revealed that the points of minimum categorization confidence (65.2%[62.9%, 67.5%]; 95% confidence interval) and minimum eatability score (67.3%[63.7%, 70.9%]) did not differ significantly from each other (*p*>.05). For the willingness to eat condition, comparisons using 95% confidence intervals indicated that the points of minimum categorization confidence (64.5%[61.9%, 67.1%]) and minimum willingness to eat score (65.1%[61.3%, 68.9%]) did not differ significantly from each other (*p*>.05).

In addition, we performed correlation analysis to examine whether the overall categorical confidence and overall eatability (or willingness to eat) scores were correlated. For the eatability condition, the results revealed a significant correlation between these indices (*r* =.96, *p*<.0001). The results for the willingness to eat condition revealed a significant correlation between these indices (*r* =.93, *p*<.0001).

Significant decreases in categorization confidence and the eatability/willingness to eat scores occurred at the same morphing rate. Moreover, categorization confidence and the eatability/willingness to eat scores were significantly correlated with each other. In other words, a higher categorization difficulty (i.e., a lower confidence in categorization) simultaneously occurred with decreases in eatability and willingness to eat. These results are consistent with our prediction and support our hypothesis that categorization difficulty for a food is related to its likability.

Yamada et al. (in press) proposed that a decrease in an object’s likability due to high categorization difficulty stems from a stranger-avoidance function of the cognitive system. That is, the cognitive system assumes a difficult-to-categorize object is a stranger (i.e., a low probability object) for agents to avoid. Based on the proposal by Yamada et al., it is predicted here that the categorization difficulty for a food will be correlated to the “likeliness” of it existing because a difficult-to-categorize food is unlikely to occur in the real world. Hence, we expected that the likeliness of existence of a food and its eatability would be correlated with each other. The next experiment was performed to test this hypothesis.

## Experiment 2

### Methods

A total of 11 subjects (six women, five men; M_age_=22.45 years, _SD_=1.92) participated in this experiment and each received a payment of Ą500 (approx. US$5.00). The subjects were naive as to the purpose of the present study, and all reported that they had normal or corrected-to-normal visual acuity.

This experiment was identical to Experiment 1 except that, in addition to eatability, the subjects were asked to evaluate the likeliness of existence of each food using a 7-point scale ranging from -3 (*definitely unlikely*) to 3 (*definitely likely*) in the evaluation task. In this experiment, willingness to eat was not measured because eatability and willingness to eat are strongly correlated and thus it seemed redundant to measure the two at the same time.

### Results and discussion

**Figure 3.**
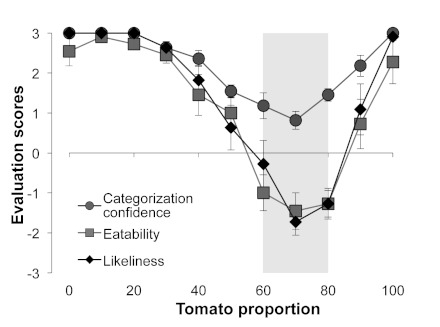
Results of Experiment 2. The gray areas indicate the tomato proportions in which all eatability, likeliness of existence, and categorization confidence values were significantly lower than those in both the tomato proportions of 0% and 100%. Error bars denote the standard errors of the mean.

Figure 3 shows the results of Experiment 2. One-way ANOVA, with the Tomato Proportion in the morphed images as a factor, performed on categorization confidence showed a significant main effect, *F*(10, 100)=22.90, *p*<.0001. Multiple comparisons using Ryan’s method revealed that categorization confidence for 50-90% images was significantly lower than for both 0% and 100% images (*p*s < .0001). One-way ANOVA on the eatability score revealed a significant main effect of the tomato proportion, *F*(10, 100)=20.46, *p*<.0001. Multiple comparisons revealed that the eatability score for 60-80% images was significantly smaller than for both 0% and 100% images (*p*s < .0001). One-way ANOVA on the likeliness of existence score revealed a significant main effect of the tomato proportion, *F*(10, 100)=29.09, *p*<.0001. Multiple comparisons revealed that the likeliness of existence score for 50-90% images was significantly smaller than for both 0% and 100% images (*p*s < .0001).

We calculated the points of minimum categorization confidence, minimum eatability and likeliness of existence scores, and their 95% confidence intervals as in Experiment 1. Comparisons using 95% confidence intervals revealed that the points of minimum categorization confidence (65.9%[63.8%, 68.0%]; 95% confidence interval), minimum eatability (69.8%[67.3%, 72.3%]), and minimum likeliness of existence (69.8%[67.2%, 72.4%]) scores did not differ significantly from each other (*p*>.05).

Correlation analysis revealed a significant correlation between the overall categorization confidences and overall eatability scores (*r* =.95, *p*<.0001), and a significant correlation between the overall categorization confidences and overall likeliness of existence scores (r =.97, *p*<.0001).

Consistent with Experiment 1, a decrease in categorization confidence coincided with a decrease in the eatability score. Moreover, we found that the likeliness of existence score was also correlated with both categorization confidence and the eatability score. These results suggest that categorization difficulty for a food is closely related to likeliness of its existence as well as to its eatability.

## Experiment 3

In Experiment 3, we investigated how food neophobia modulates the effect of categorization difficulty on food likability. For this purpose, we employed a food neophobia scale (Imada & Yoneyama, 1998) to measure the degree of individuals’ food neophobia traits. This scale was developed for testing food neophobia in Japanese people, based on the original scale of Pliner and Hobden (1992).

### Methods

Sixty-one college students participated in this experiment in exchange for course credits. Data from subjects with more than one missing value were excluded from further analysis, and hence data from 52 subjects (15 women, 36 men, and one unknown; M_age_=20.39 years, *SD*=3.00) were finally analyzed. The subjects were naive as to the purpose of the present study.

In this experiment, data collection was paper-based.^2^ The images used in the previous experiments were printed in color on paper. Rating items were positioned below each image. Three items were employed: eatability, likeliness of existence, and categorization confidence. A 7-point scale was used as in the previous experiments. A Japanese version of the food neophobia scale was also used at the same time. This scale consisted of 14 items that asked subjects for their attitudes regarding food neophobia, for example, “I fear eating novel foods” and “I want to try new food products.” The subjects were jointly tested in one room and there was no time limit for their responses.

### Results and discussion

We computed Cronbach’s alpha of the food neophobia scale scores (α = .82), showing a high internal consistency for the scale. Based on the food neophobia scale score, we divided the subjects into the high and low food-neophobia groups using a median split. A two-tailed, two-sample t test revealed a significant difference in the food neophobia scale scores between the high and low food-neophobia groups, *t*(50)=8.36, *p*<.0001.

**Figure 4.**
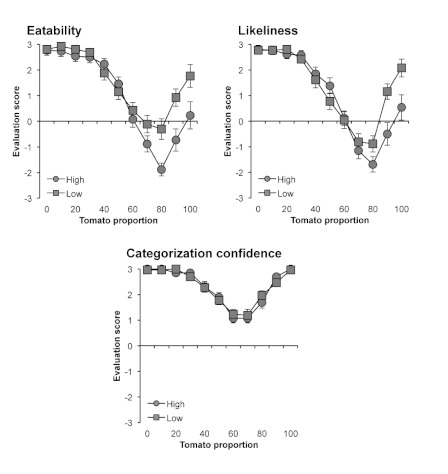
Results of high and low food neophobia groups in Experiment 3. The results of eatability, likeliness of existence, and categorization confidence are shown separately. Error bars denote the standard errors of the mean.

Figure 4 shows the results of Experiment 3. Mixed ANOVA, with Food Neophobia (high or low) as a between-subject factor and the Tomato Proportion in the morphed images as a within-subject factor, performed on the eatability score showed significant main effects of food neophobia, *F*(1, 50)=4.65, *p*<.05, and tomato proportion, *F*(10, 500)=65.09, *p*<.0001, and a significant interaction, *F*(10, 500)=4.39, *p*<.0001. Post-hoc tests revealed a significant simple main effect of food neophobia for 80-100% images (*p*s<.001). Multiple comparisons using Ryan’s method revealed that the eatability score for 60-90% images was significantly lower than for both 0% and 100% images (*p*s<.0001).

Mixed ANOVA on the likeliness-of-existence score showed a significant main effect of tomato proportion, *F*(10, 500)=74.34, *p*<.0001, and a significant interaction, *F*(10, 500)=4.44, *p*<.0001. However, no main effect of food neophobia was found, *F*(1, 50)=1.80, *p*=.18. Post-hoc tests revealed a significant simple main effect of food neophobia for 80-100% images (*p*s<.05). Multiple comparisons revealed that the likeliness-of-existence score for 60-90% images was significantly lower than for both 0% and 100% images (*p*s<.0001).

Mixed ANOVA on categorization confidence showed a significant main effect of tomato proportion, *F*(10, 500)=57.33, *p*<.0001. However, no main effect of food neophobia, *F*(1, 50)=0.002, *p*=.97, and no interaction were found, *F*(10, 500)=0.63, *p*=.79. Multiple comparisons revealed that the categorization confidence for 40-80% images was significantly lower than for both 0% and 100% images (*p*s<.0001).

As in the previous experiments, as categorization difficulty increased, judged eatability and likeliness of existence decreased. The result patterns for eatability and likeliness of existence were slightly different between the high and low food-neophobia groups, while the pattern for categorization confidence was statistically equivalent between them, suggesting that food neophobia is a factor that modulates food likability while keeping categorization difficulty intact.

## General discussion

The present study was performed to examine whether categorization difficulty of a food based on its appearance is related to food likability. We presented the subjects with stimulus images created by morphing tomato and strawberry photographs and asked them to categorize the food in each image and to evaluate eatability or willingness to eat as indices of food likability. In Experiment 1, lower confidence in categorization of a food coincided with lower evaluations of eatability and willingness to eat, suggesting that categorization difficulty of food was strongly related to food likability. In Experiment 2, categorization difficulty was also related to likeliness of existence of a food. In Experiment 3, individual difference in food neophobia was a factor that modulated the effect of categorization difficulty on food likability and likeliness of existence of a food.

These results suggest that the effect of categorization difficulty on object likability is not specific to living stimuli. A previous study showed that categorization difficulty was related to likability regarding human and dog faces (Yamada et al., in press). Considering a previous finding on the dissociation of cognitive processing of living and non-living things (Forde & Humphreys, 2002), it was suggested that the effect of categorization difficulty on likability may be specific to living stimuli (i.e., human and dogs). However, this is not the case. The three experiments in the present study showed that categorization difficulty was closely related to food likability.

It is plausible that the relationship identified between categorization difficulty and eatability/willingness to eat is evolutionarily adaptive. As described above, Yamada et al. (in press) interpreted their results by introducing the concept of “stranger avoidance.” This account was based on the assumption that human cognitive systems tend to avoid organisms that are potentially harmful (Zajonc, 1968). In particular, the harmfulness of organisms that are difficult to categorize (i.e., strangers) is evaluated as high, leading to avoidance reactions toward strangers. In a similar vein, it is likely that the negative evaluation for foods that are difficult to categorize can be explained in terms of “strange food” avoidance. Strange foods can be hazardous to biological organisms or genes, and hence the cognitive system may block the ingestion of such foods by invoking negative impressions. The results of Experiment 2 support this suggestion in so far as unlikely foods are always strange and therefore such foods are judged to be not eatable.

Food neophobia is not a product of categorization difficulty but a factor in the modulation of food likability. In Experiment 3, food neophobia did not change the pattern of results for categorization confidence, whereas food neophobia did affect food eatability and likeliness of existence. Previous research has suggested that food neophobia is influenced by some factors that are not directly related to categorization difficulty (Martins et al., 1997; McFarlane & Pliner, 1997; Pelchat & Pliner, 1995; Pliner et al., 1993; Tuorila et al., 1994). Taken together with our findings, these observations suggest that food neophobia is based on information processing other than object categorization, and it affects the likability of foods independently of categorization difficulty.

It is of interest to note that visual information related to foods may be linked to the mental imagery of their taste, and that the mental imagery of food taste affects food likability. A previous study showed that the vividness of the mental imagery of a food’s taste, as well as its appearance, is positively correlated with food cravings (Tiggemann & Kemps, 2005). Other research indicated that instructions on taste information, such as “it tastes good,” diminished avoidance responses to novel foods (Martins et al., 1997; Pelchat & Pliner, 1995). Similarly, it is likely that when subjects can clearly imagine the taste of foods, they will be willing to eat those foods. Based on these previous findings, it is likely that food likability is related to the clarity of mental imagery for taste, which is based on the difficulty of visual categorization or likeliness of existence of the foods. Future studies may address this issue by combining the methods used in the present study with measurements of mental imagery regarding the taste of foods.

## Footnotes

1 We used the two measures (eatability and willingness to eat) in separate subject groups in Experiment 1 to show that the effect of categorization difficulty is not dependent on one specific measure in one sample group. Eatability and willingness to eat seem to measure a similar concept related to food likability, and hence it was predicted that the results of these measures would show a similar pattern.

2 We used paper-based data sampling in Experiment 3 because we needed a larger sample than in the previous experiments to investigate individual differences in food likability and food neophobia. Regardless of the difference in procedure, essentially similar results were obtained across the experiments. However, there was an unexpected effect in Experiment 3: The points with the lowest score shifted toward the tomato category. This may have been due to visual degradation of the stimuli caused by printing. However, the shift in score did not affect our main conclusion drawn from the previous experiments. Thus, we believe that there were essentially no differences between the results obtained from computerized and paper-based procedures.

## Author note

Yuki Yamada, Takahiro Kawabe, and Keiko Ihaya contributed equally to the research and publication of the study.
